# Cortical Gyrification Patterns Associated with Trait Anxiety

**DOI:** 10.1371/journal.pone.0149434

**Published:** 2016-02-12

**Authors:** Tara A. Miskovich, Walker S. Pedersen, Emily L. Belleau, Skyler Shollenbarger, Krista M. Lisdahl, Christine L. Larson

**Affiliations:** Department of Psychology, University of Wisconsin-Milwaukee, Milwaukee, WI, United States of America; Institute of Psychology, Chinese Academy of Sciences, CHINA

## Abstract

Dispositional anxiety is a stable personality trait that is a key risk factor for internalizing disorders, and understanding the neural correlates of trait anxiety may help us better understand the development of these disorders. Abnormal cortical folding is thought to reflect differences in cortical connectivity occurring during brain development. Therefore, assessing gyrification may advance understanding of cortical development and organization associated with trait anxiety. Previous literature has revealed structural abnormalities in trait anxiety and related disorders, but no study to our knowledge has examined gyrification in trait anxiety. We utilized a relatively novel measure, the local gyrification index (LGI), to explore differences in gyrification as a function of trait anxiety. We obtained structural MRI scans using a 3T magnetic resonance scanner on 113 young adults. Results indicated a negative correlation between trait anxiety and LGI in the left superior parietal cortex, specifically the precuneus, reflecting less cortical complexity among those high on trait anxiety. Our findings suggest that aberrations in cortical gyrification in a key region of the default mode network is a correlate of trait anxiety and may reflect disrupted local parietal connectivity.

## Introduction

Dispositional anxiety is a stable personality trait with biological underpinnings [1] that is a risk factor for numerous psychiatric disorders, including anxiety disorders, depression, and substance use [2–4]. Understanding individual differences in brain structure associated with this personality trait has the potential to advance understanding of the etiology of these burdensome disorders.

Previous research has shown that trait anxiety and anxiety-related traits are associated with variation in cortical volume and thickness of regions implicated in anxiety-related processes. For instance, trait anxiety and neuroticism have both been linked to reduced thickness in the orbitofrontal cortex [1,5,6]. Behavioral inhibition, a construct similar to trait anxiety, has been associated with decreased gray matter volume in the medial orbitofrontal cortices and the precuneus [7]. The orbitofrontal cortex is important for various cognitive and emotional processes that are thought to be impaired in anxiety, such as extinction learning [8]. Additionally, both the orbitofrontal cortex and precuneus have been identified as nodes in the default mode network (DMN), a neural network involved in self-referential or self-focused attentional processing [9–11]. This network is particularly relevant for internalizing disorders such as anxiety and depression, as DMN dysfunction may be a core neural substrate of pathological self-focused thought, such as rumination or worry [12–14].

As these studies indicate, trait anxiety is associated with abnormalities in cortical volume and thickness; however, to our knowledge no one has assessed the relationship between trait anxiety and cortical folding or gyrification. The process of gyrification begins in the second trimester in utero [15,16], and is thought to be largely complete by the third trimester [17]. Gyrification has been posited to reflect underlying structural connectivity because cortical folding allows for more efficient network wiring as surface area expands in the rapidly developing brain [18]. Initially, theories of gyrification argued the ratio of folding to surface area (the gyrification index) [19] remains relatively constant after the first few years of life, and therefore should primarily reflect early developmental, and genetic influences [17]. However, more recent evidence has pointed to developmental changes in gyrification throughout adolescence and adulthood [16,20–22]. Additionally, monozygotic twins have differences in gyrification patterns, suggesting a greater role for environmental factors influencing gyrification, more so than measures of cortical volume [23,24]. This is particularly true within more superficial sulci [25]. Therefore, differences in gyrification patterns may arise across a larger window of development than previously thought. Importantly, gyrification has been linked to measures of cortical connectivity, both functional [26] and structural [27]. Thus, abnormal cortical folding may provide insight into variation in cortical connectivity that may arise during cortical development.

Aberrant gyrification has been linked to various psychiatric disorders such as schizophrenia [28,29] and autism [30,31]. Abnormal morphology may, therefore, represent a disruption in neurodevelopment that puts one at risk for these and other disorders. Of most relevance for the current study, investigations across clinical samples of anxiety and depression in adults have revealed gyrification abnormalities in the precuneus and posterior cingulate, another key node in the posterior DMN [32–34]. Additionally, there is some evidence suggesting that gyrification abnormalities are linked to dysfunction in this network. Nixon and colleagues [33] found that individuals with depression displayed hypogyrification in the precuneus, as well as increased task-based functional connectivity between the precuneus and anterior nodes of the DMN. Since the DMN is thought to subserve self-focused thought, excessive connectivity may reflect pathological self-focus such as worry [13,14]. Thus, research in this area suggests that there may be a link between gyrification and DMN dysfunction in internalizing disorders that may reflect a vulnerability to developing an anxiety or depressive disorder.

The aim of the current study was to investigate abnormalities in cortical folding that may be associated with trait anxiety, a core risk factor for some of the most prevalent forms of psychopathology, including anxiety disorders, depression, and substance use. Examining gyrification alongside trait anxiety may provide us with important clues about structural differences that arise during neurodevelopment that are associated with this important risk factor. We used a three-dimensional measurement of gyrification, the local gyrification index (LGI) [35], to assess differences in gyrification as a function of trait anxiety.

## Materials and Methods

### Participants

One hundred and twenty one individuals ranging from 18 to 35 (72 females) were recruited from the University of Wisconsin-Milwaukee student body and local community to participate in the study. All aspects of this study were approved by both the Medical College of Wisconsin and the University of Wisconsin, Milwaukee (IRB# PRO00012620). Informed written consent was obtained from each participant in accordance with the University of Wisconsin-Milwaukee and Wisconsin Medical College Institutional Review Boards. Mean age of the sample was 21.9 ± 3.8 years. Subjects were excluded from the study if they were left handed, had a history of significant head trauma, contradiction to magnetic resonance imaging, history of a neurological disorder, psychotic disorder, or bipolar disorder, as assessed by the Structured Clinical Interview for DSM-IV Disorders [36].

Eight subjects were excluded from the final analyses. Six were dropped due to excessive motion during scanning, one was dropped due to technical issues with the scanner, and one was dropped due to the presence of manic episodes. Final data analyses included the remaining 113 subjects.

### Quantifying Trait Anxiety

Participants completed the Trait version of the Spielberger State-Trait Anxiety Inventory (STAI) [37], a 20-item questionnaire that has high test-retest reliability as well as high internal consistency [38]. The mean STAI score was 39.7 ± 10.7 (range 21–66).

### MRI Acquisition

High resolution spoiled gradient recalled (SPGR) images were acquired in a sagittal orientation (TR = 8.2ms; TE = 3.2ms; FOV = 24cm; flip angle = 12°; voxel size (0.9375 x 0.9375 x 1mm) on a 3.0 Tesla short bore GE Signa Excite MRI system using a 12-channel head coil.

### Cortical Reconstruction and LGI Maps

FreeSurfer 5.0 (www.nmr.mgh.harvard.edu/freesurfer) [39,40] installed on a Red Hat Enterprise Linux v.6.6 was used to reconstruct the cortical surface as a triangular mesh. The Freesurfer reconstruction pipeline is described in more detail in [39], but consists of transformation to Talairach space, normalization of intensity, removal of non-brain tissue (skull), and segmentation of gray and white matter. In addition, we used manual editing to further correct the pial and white matter surfaces and remove skull and dura mater left over from skull-stripping.

The LGI, developed by Schaer and colleagues [35], was used to assess the ratio of buried to visible cortex, or amount of gyrification. This technique is a three-dimensional extension of the GI technique of Zilles and colleagues [19]. This new method is fully automated, and therefore provides a less subjective measure that takes into account the three-dimensional nature of the cortical folds and is not biased by the orientation of the slices [35]. In addition, the primary advantage is the ability to localize specific abnormalities in cortical folding, whereas previous methods only yielded a global measure of gyrification. The FreeSurfer LGI computation creates a triangulated mesh of the outer hull of the brain, calculates the LGI ratio between the pial surface and outer hull at the center of a 25 mm spherical region of interest, and propagates these values onto the pial surface, assigning LGI values to each vertex on the mesh surface [35,41].

### Statistical Analyses

The relationship between trait anxiety and gyrification was assessed on surface maps at every vertex in the brain using the different offset, different slope (DODS) design provided in Qdec in FreeSurfer, with age and sex added as covariates. LGI values were first mapped to a normalized brain for each subject. We applied a smoothing kernel of 5 mm FWHM. To correct for multiple comparisons cluster correction was done using Monte Carlo simulation with 10,000 iterations. Using a vertex-wise threshold of *p* < .05, a minimum cluster size of 688.85 mm^2^ was considered significant.

For clusters showing a significant relationship between LGI and trait anxiety, we extracted average LGI, along with surface area and cortical thickness. Although these measurements have been linked to gyrification [20,21], they have different developmental trajectories [42], thus, examining these measurements jointly may provide a more comprehensive account of structural differences related to trait anxiety. Cortical thickness was defined as the distance between the gray and white matter boundary. This distance was calculated at each vertex and then averaged within significant clusters. Surface area was calculated by assigning the average area of surrounding triangles to each vertex and summing those values within significant clusters to obtain total surface area for the cluster. Multiple regressions, conducted in SPSS, were used to determine the relationship between LGI and surface area, cortical thickness, and trait anxiety. Age and sex were used as covariates in these analyses, and the results are presented in partial correlations.

## Results

**Fig 1:**
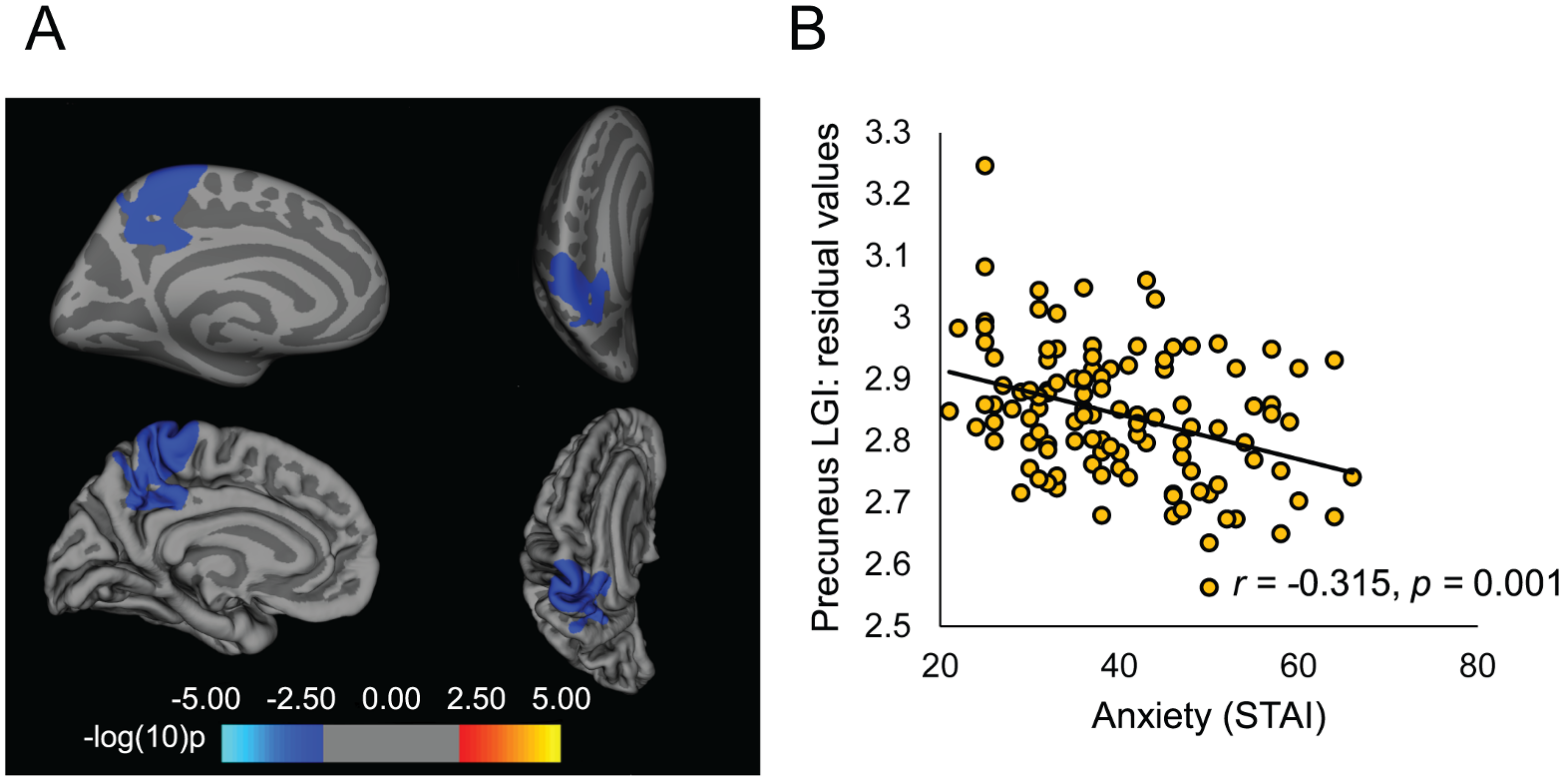
Decreased precuneus gyrification is associated with trait anxiety. (A) Inflated and pial surface maps of the left hemisphere demonstrating decreased gyrification in the precuneus as a function of trait anxiety. Images on the left depict the medial view of the left hemisphere. Images on the right are a view from the top of the right hemisphere and are tilted 30 degrees to provide a better angle for viewing the cluster extent. (B) Scatterplot of the correlation between trait anxiety and average LGI within the precuneus cluster.

The vertex-wise analysis revealed a negative correlation between trait anxiety and LGI in the precuneus in the left hemisphere (cluster size = 2608.73 mm^2^, 5940 vertices, cluster corrected *p* = .003; Fig 1). There were no regions where LGI was positively associated with trait anxiety, and there was no moderating effect of sex. There was no relationship between anxiety and gyrification in the right hemisphere.

**Fig 2:**
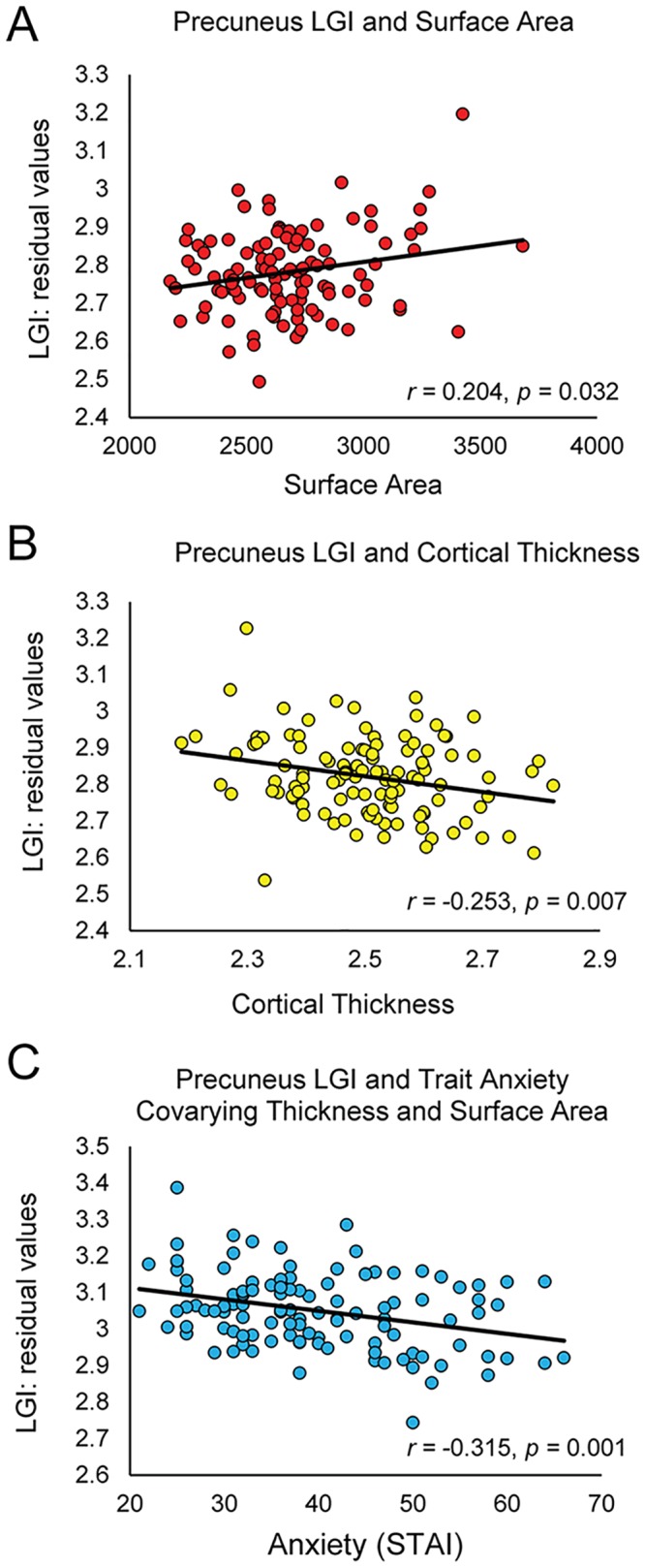
Correlations between LGI and other cortical measurements. (A) Scatterplot of the correlation between total surface area and average LGI within the precuneus. (B) Scatterplot demonstrating a negative correlation between average cortical thickness and average LGI within the precuneus. (C) Scatterplot demonstrating the correlation between trait anxiety and LGI when controlling for both cortical surface area and thickness.

We conducted follow up analyses to better understand how other features of the cortical surface may be related to or explain differences in gyrification in the precuneus. Specifically, we correlated average surface area and cortical thickness with average LGI within the significant precuneus cluster. Controlling for the effects of sex and age, we found that surface area was positively associated with LGI (*r* = 0.204, *p* = 0.032; Fig 2A). Additionally, we found that cortical thickness was negatively correlated with LGI (*r* = -0.253, *p* = 0.007; Fig 2B). The relationships between these measurements and gyrification are consistent with previous findings [20,21]. To determine whether differences in surface area and cortical thickness accounted for the relationship between LGI and trait anxiety, we assessed the relationship between average precuneus LGI and trait anxiety with average surface area and cortical thickness added as covariates (in addition to age and sex). We found that even after controlling for variance in surface area and cortical thickness, there was still a negative association between trait anxiety and LGI (*r* = -0.315, *p* = 0.001; Fig 2C).

## Discussion

In the current study, we investigated differences in cortical gyrification associated with dispositional anxiety. We found that there was less gyrification in the left precuneus as dispositional anxiety increased. Although both cortical thickness and surface area were associated with gyrification, the relationship between precuneus gyrification and trait anxiety remained significant when controlling for these other cortical measurements. These findings suggest that there may be differences that arise during neurodevelopmental in the precuneus in those with dispositional anxiety, though longitudinal replication is warranted. Since theoretical [43] and empirical work [26,27,33] have linked gyrification to underlying cortical connectivity, these aberrations in cortical morphology may be indicative of abnormalities in connectivity between the precuneus and corresponding networks.

Functionally, the precuneus is involved in broad attentional and memory processes, as well as self-reflection and self-focused thinking [44], and is a key node in the DMN [9–11]. Studies of anxiety and anxiety disorders have found abnormalities in both activation and functional connectivity of the DMN [45]. Increased DMN activation has been also been linked to subjective reports of worry [13,14]. Additionally, several studies have found that in healthy individuals, precuneus activation is negatively correlated with activation of the amygdala [46,47]. Moreover, connectivity between these regions has also shown to be altered in social anxiety [48], panic disorder [49] and in youths with generalized anxiety disorder [50]. Thus, the precuneus may play a modulatory role in affective processing, which is disrupted in anxiety [7,47]. The current findings raise the possibility that structural abnormalities in the precuneus may underlie some of the functional abnormalities previously observed in anxiety in this region and related circuitry.

Our findings are consistent with previous studies showing precuneus structural abnormalities in anxiety-related personality dimensions and LGI-specific precuneus findings in clinical populations. Both behavioral inhibition [7] and harm avoidance, [51] have been associated with decreased precuneus gray matter volume. This relationship has also been detected in trait anxiety, but did not survive correction for multiple comparisons [52]. Additionally, although this is the first study to assess gyrification within dispositional anxiety, our findings are consistent with previous studies examining gyrification in clinical samples of anxiety and depression. Yoon and colleagues [32] demonstrated that hypogyrification in the precuneus was associated with greater severity of symptoms in panic disorder, and Zhang and colleagues found precuneus/posterior cingulate cortex (PCC) hypogyrifaction in individuals with depression. Nixon and colleagues [33] replicated the finding of decreased precuneus gyrification in depression and further showed that this hypogyrification was associated with hyper-connectivity between the precuneus and the dorsal lateral prefrontal cortex. Their findings are consistent with the hypothesis that morphological abnormalities may reflect altered functional connectivity. The present study extends these previous findings by suggesting that abnormalities in precuneus cortical folding across these disorders may in part be due to the presence of these abnormalities in dispositional anxiety, a core risk factor that is common to these emotional disorders [2–4].

As posited above, the current findings may provide insight into the development of trait anxiety by demonstrating a structural correlate that may arise during neurodevelopment. Although earlier theories posit that the degree of brain gyrification takes place primarily in utero and then stays constant through childhood and beyond [16,17], recent evidence has suggested that there are developmental changes in gyrification throughout the lifespan [16,20–22]. Since the age of onset for various anxiety disorders ranges from early childhood to adulthood [53], it is hard to definitely understand when these cortical differences occur without assessing changes longitudinally. Additionally, the current study can only infer that these patterns may represent abnormalities in neurodevelopment, for we did not assess cortical changes across development or between individuals of different ages. However, structural abnormalities in the precuneus and posterior cingulate have been identified in pediatric samples, with anxious youths showing gray matter volume abnormalities [54,55], supporting the possibility that the current LGI findings represent aberrations in early neurodevelopment.

We also investigated how other aspects of cortical structure correlated with LGI in the precuneus in order to provide a broader picture of the cortical surface and better understand how these measurements are related to each other. Consistent with previous research [20,21], surface area had a positive relationship with LGI while cortical thickness had a negative relationship. While LGI was associated with thickness and surface area, these variables did not fully account for the relationship between LGI and anxiety. Since other cortical structural measures could not fully explain the relationship between LGI and trait anxiety, it is possible that other structural factors are influencing this relationship. For instance, certain theories of gyrification have emphasized the role of white matter tension [43] or white matter constraints [56] in the development of gyrification. Therefore, it is possible that the current findings reflect underlying differences in white matter strength. Further research on gyrification may be useful in understanding the association between gyrification abnormalities and the structural connectivity of broader neural networks in this population.

## Conclusions

In conclusion, we found differences in cortical gyrification associated with trait anxiety that could not be explained by surface area or thickness within a key DMN node. These findings are consistent with findings in clinical populations, highlighting the possibility that differences in local parietal connectivity are associated with a personality dimension that is a key risk factor for many of the most common forms of psychopathology. Although we did not investigate connectivity directly, future research may shed light on whether these structural differences are linked to disruptions in network connectivity. This structural aberration may in turn contribute to the emotional difficulties evident in high trait anxiety through influences on a primary node of the DMN subserving self-referential and self-conscious thought.
